# Serious infectious events and ibuprofen administration in pediatrics: a narrative review in the era of COVID-19 pandemic

**DOI:** 10.1186/s13052-021-00974-0

**Published:** 2021-01-29

**Authors:** Lucia Quaglietta, Massimo Martinelli, Annamaria Staiano

**Affiliations:** 1 Santobono-Pausilipon Children’s Hospital, Naples, Italy; 2 Department of Translational Medical Science, Section of Pediatrics, University of Naples “Federico II”, Via S. Pansini, 5, 80131 Naples, Italy

**Keywords:** Chickenpox, COVID19, Cystic fibrosis, Ibuprofen, Pediatrics, Pneumonia, Sepsis

## Abstract

**Purpose of review:**

Despite its recognized efficacy and tolerability profile, during the last decade a rise of adverse events following ibuprofen administration in children has been reported, including a possible role in worsening the clinical course of infections. Our aim was to critically evaluate the safety of ibuprofen during the course of pediatric infectious disease in order to promote its appropriate use in children.

**Recent findings:**

Ibuprofen is associated with severe necrotizing soft tissue infections (NSTI) during chickenpox course. Pre-hospital use of ibuprofen seems to increase the risk of complicated pneumonia in children. Conflicting data have been published in septic children, while ibuprofen in the setting of Cystic Fibrosis (CF) exacerbations is safe and efficacious. No data is yet available for ibuprofen use during COVID-19 course.

**Summary:**

Ibuprofen should not be recommended for chickenpox management. Due to possible higher risks of complicated pneumonia, we suggest caution on its use in children with respiratory symptoms. While it remains unclear whether ibuprofen may have harmful effects during systemic bacterial infection, its administration is recommended in CF course. Despite the lack of data, it is seems cautious to prefer the use of paracetamol during COVID-19 acute respiratory distress syndrome in children.

## Introduction

Ibuprofen belongs to the non-steroidal anti-inflammatory drugs (NSAIDs) and based on the most recent international guidelines is the currently recommended antipyretic and analgesic to be used in pediatric age together with paracetamol [1–3]. Its effectiveness to relieve pain and reduce fever discomfort is widely demonstrated by several clinical trials [4–9]. Despite its commonly recognized efficacy and tolerability profile, starting from 2010 the Pediatric Working Group of the Italian Drugs Agency (AIFA) reported an increase of suspected adverse reactions possibly related to ibuprofen use in parallel with its growing over-the-counter consumption [10]. As a matter of fact, during the last decade a worrying rise of papers describing adverse events occurring in children under ibuprofen and other NSAIDs therapy have been published [11–14]. The main reported side effects seem to involve the gastrointestinal system [11, 12] and the kidneys especially in feverish dehydrated individuals [13, 14]. Nevertheless, a possible role of NSAIDs in worsening the clinical course of bacterial as well as viral infections has also been suspected for decades, especially for skin and soft tissue infections (SSTI) [15]. In 2009 Legras et al. conducted a multicenter case-control study in order to establish whether the use of NSAIDs in the course of bacterial community-acquired infections in adults was associated with severe sepsis or septic shock [16]. Although the use of NSAIDs in patients with severe sepsis or septic shock did not differ from those with mild infection at the same infected site, a longer median time of antibiotic therapy was observed in NSAIDs’ users [16]. Nevertheless, the impact of NSAIDs intake during bacterial infections remains controversial [15]. In this scenario, in April 2019 the French National Agency for the Safety of Medicines and Health Products (ANSM) issued a warning about the use of NSAIDs for patients with infectious diseases based on the analysis of 20 years of real-world safety data of ibuprofen and ketoprofen [17]. The analysis included 337 and 49 cases, respectively, over 20 years of infectious complications. Most of the complications were related to *Streptococcus* and occurred within 2 or 3 days from the starting of NSAIDs’ therapy [17]. In some cases, NSAIDs were administered concomitantly with antibiotics and in many cases without medical advice [17]. Following this warning, the ANSM released practical recommendations on NSAIDs use inviting to limit NSAIDs consumption at the minimal effective dose and for the shortest possible time [18, 19]. In details, treatment should be continued for no more than 3 days for fever and 5 days for pain and discontinued at symptoms resolution. Patients were advised not to assume more than one type of NSAIDs at a time [18, 19]. ANSM also stated that the use of NSAIDs has to be considered contraindicated in cases of chickenpox [20]. The exact mechanism on how NSAIDs might affect the pathogenesis of complicate infectious diseases is still unclear. It has been postulated that NSAIDs may mask the signs and symptoms of bacterial infection, thus delaying appropriate treatment [21–24]. NSAIDs may also modify the host inflammatory response both promoting neutrophils influx and inhibiting cytokine/interleukin/tissue necrosis factor production, thus creating a more suitable environment for bacterial growth [21]. Finally, it is postulated that fever itself has an important role in infection control and NSAIDs mediated fever suppression may interfere with the host control of viral and bacterial infections [21].

In light of the emerging evidences, the aim of this review was to critically evaluate the safety of ibuprofen during the course of pediatric infectious disease in order to highlight circumstances associated with higher risks and to promote safe and appropriate use of this drug in children.

## Methods

A literature search was performed including Medline-PubMed database, using April 20th as last search date. We matched the terms (Newborn OR Infant OR Child OR Adolescent OR Pediatrics) AND (Ibuprofen OR NSAIDs OR Non-steroidal anti-inflammatory drugs OR Anti-inflammatory) AND (Chickenpox) OR (Pneumonia OR Community acquired pneumonia OR Lower respiratory tract infection OR LRTI OR Upper respiratory tract infection OR URTI) AND (Empyema OR Pleural effusion OR Pleural empyema OR Parapneumonic effusion) AND (Sepsis) AND (Skin Infections), AND (COVID-19).

### Chickenpox

Chickenpox is a highly contagious, common epidemic disease in young children, with 90% of infections before the age of 10 years old and median onset of the disease at 3 years old [25]. The risk of chickenpox complications may be significantly increased after NSAIDs’ exposure. In particular, NSAIDs are able to promote the development of bacterial super-infection, to mask symptoms and to cause delay management. Table 1 summarizes the data on ibuprofen use in children with chickenpox. One of the most common complications of chickenpox is represented by skin super-infections, mainly caused by group A streptococcal (GAS) infections, which are also responsible for necrotizing fasciitis (a rapidly progressive inflammatory infection of the fascia) with secondary necrosis of the subcutaneous tissues. A strong association between the use of NSAIDs and SSTI complications (mostly cellulitis and abscess) in children with chickenpox has been previously reported [23, 26]. Lesko et al. investigated the risk factors implicated in the development of necrotizing fasciitis, analyzing 224 subjects with chickenpox, of whom 52 with GAS infection and 172 with uncomplicated chickenpox [27]. Among the 224 children, 123 had taken ibuprofen or paracetamol (alone or in combination). The authors found that the use of ibuprofen was not associated with a higher risk of developing soft tissue necrosis, while the probability of a GAS infection was higher in subjects who had taken ibuprofen alone (OR 3.9, 95% CI: 1.3–12) [27]. More recently, Mikaeloff et al. conducted a 12-year epidemiological case–control study based on a cohort of 140,111 individuals with chickenpox diagnosis, to determine whether NSAIDs could increase the risk of severe skin complications [28]. Despite some potential biases related to the NSAIDs exposure, the authors concluded that the use of NSAIDs was associated with an increased risk of skin and soft tissue complications in the context of chickenpox, especially in children [28]. Souyri et al. evaluated the contribution of NSAIDs to the development of severe necrotizing soft tissue infections (NSTI) comparing 38 subjects with NSTI with 228 matched healthy controls [29]. The 38 subjects with NSTI were divided into three groups on the basis of age: 12 infants (0–23 months), 16 children (2–15 years) and 10 adults (> 15 years). Of the 38 patients with NSTI, 25 patients were exposed to ibuprofen (OR 31.38; 95% CI 6.40–153.84) and 24 presented with chickenpox (OR 17.55; 95% CI 3.47–88.65). This study indicates a strong association between the use of NSAIDs and severe NSTI, in particular in children with chickenpox [29]. As a consequence of these growing evidences, the use of ibuprofen for symptom control in chickenpox has been progressively abandoned [30, 31].

**Table 1:** Ibuprofen and chickenpox

Year	1st author (Ref)	Children (N)	Disorder	Treatment	Adverse events
1997	Choo [26]	7865	Chickenpox	Ibuprofen	SSTI
1999	Zerr [23]	48	Chickenpox	Ibuprofen	Necrotizing fascitis
2001	Lesko [27]	224	Chickenpox	Ibuprofen +/−	GAS infections and
				paracetamol	soft tissues necrosis
2008	Mikaeloff [28]	140,111	Chickenpox	Ibuprofen	Cellulitis, abscess,
				(or other NSAIDs)	fasciitis and necrosis
2008	Souyry [29]	266	Chickenpox	Ibuprofen	Serious necrotising
			and other	(or other NSAIDs)	soft-tissue infections
			viral infections		
2008	Dubos [30]	159	Chickenpox	Ibuprofen	Secondary bacterial
					skin complications

*GAS* Group A streptococci, *N* Number of enrolled children, *NSAIDs* Non-steroidal anti-inflammatory drugs, *Ref* Reference number, *SSTI* Skin and soft tissue infection

In conclusion, current evidences clearly underline a concrete risk of NSTI in children assuming ibuprofen during the course of chickenpox. Therefore, its use has to be strongly discouraged for the management of chickenpox related symptoms in children.

### Sepsis

Sepsis is a life-threatening organ dysfunction determined by a dysregulated host response to infections [32]. A number of case reports, concerning patients admitted to intensive care units, suggested that the use of NSAIDs might increase the severity of bacterial infections leading to shock and multiple organ failure [33–36]. This seems to be consequent to life-threatening infections, mainly streptococcal, such as streptococcal toxic shock syndrome (STSS) or necrotizing fasciitis, but also driven by other organisms such as Staphylococcus spp. or Gram-negative bacilli [36–39]. Several investigations reported a sequential relationship between the administration of NSAIDs and the progression of invasive GAS infections [35, 38, 40–43]. Nevertheless, up to date, few studies have been published in children with conflicting results [44–46]. An epidemiologic study from the UK found that STSS was independently associated with NSAIDs use with a 3-fold increase, including children (OR: 3; 95% confidence interval, 1.30–6.93; *p* = 0.01) [44]. However, as well underlined by the authors, no data was collected regarding time, dose, indications, and specific NSAIDs used, making very difficult to draw any conclusion [44]. On the other hand, NSAIDs, particularly ibuprofen, have been used in most studies for the treatment of sepsis. In 1997 a randomized, double blind, placebo-controlled trial on adults conducted by Bernard et al. demonstrated that the group treated with ibuprofen showed decreased levels of prostacyclin and thromboxane as well as a quicker resolution of fever, tachycardia, oxygen consumption and lactic acidosis. However, the mortality rate by day 30 did not differ significantly in the ibuprofen and placebo groups (37% vs. 40%). No differences in terms of survival were observed between the 2 groups [45]. More recently, in 2012 Demirel et al. observed that sepsis parameters in preterm infants with patent ductus arteriosus (PDA) decreased after ibuprofen administration independently from antibiotic therapy [46]. Table 2 sums up the evidences highlighting the relationship between ibuprofen administration and sepsis in children.

**Table 2:** Ibuprofen exposure and sepsis in children

1st Author, Year (Ref)	Population; Country	Study Design	Outcome	Key results	Comments
*Bernard, 1997* [45]	455 children with sepsis, defined as fever, tachycardia, tachypnea, and acute failure of at least one organ system.	Randomized, double-blind, placebo-controlled trial	Mortality rate at 30 days from the sepsis	Mortality by day 30 did not differ significantly in the ibuprofen and placebo groups (37% vs 40%). In the ibuprofen group, there were significant declines in urinary levels of prostacyclin and thromboxane, temperature, heart rate, oxygen consumption, and lactic acidosis.	None
*Lamagni, 2008* [44]	2607 adults and 318 children with severe *Streptococcus pyogenes* infection	Prospective, observational, study	STSS development	Patients who used NSAIDs had a 3-fold increased risk for STSS (OR 3.00, 95% CI 1.30–6.93, *p* = 0.01).	No data collected regarding time, dose, indications, and specific NSAIDs used.
*Demirel, 2012* [46]	51 preterm infants with sepsis treated with Ibuprofen for PDA versus 38 preterm infants with sepsis without PDA not treated with Ibuprofen	Prospective, observational, study	Effects on the production of IL-6 and CRP	CRP and IL6 were significantly decreased at day 4th or 5th and 7th or 10th after sepsis diagnosis in the group of children treated with Ibuprofen for PDA (*p* = 0.02, *p* = 0.01, *p* = 0.03, p = 0.01, respectively)	Non-interventional study, with unclear exclusion criteria and relatively small sample size.

*CRP* C-reactive protein, *NSAIDs* Non-steroidal anti-inflammatory drugs, *PDA* Patent ductus arteriosus, *STSS* Streptococcal toxic shock syndrome

In conclusion, it remains unclear whether NSAIDs may have harmful effects during the course of systemic bacterial infection. We highlight the need for well-designed pediatric trials in order to better define the relative risks and benefits of NSAIDs administration in this delicate setting.

### Pneumonia

In order to investigate a possible causality between the intake of NSAIDs and the development of severe lung bacterial infections in childhood, Leroy et al. analyzed a prospective cohort of 5182 hospitalized children over a 3-years’ period [47]. Of these, 32 (0.6%) had severe bacterial infections following treatment with NSAIDs, mainly ibuprofen, in the 15 days prior to the admission. Bacteriological studies identified the presence of *Staphylococcus aureus*, GAS and *Streptococcus pneumoniae* [47]. In US a retrospective study reported an increasing incidence of pleural empyema between 1993 and 1999, rising from one to five cases per 100,000 people aged less than 19 years old. Among children hospitalized for Community Acquired Pneumonia (CAP) during the study period, 28% developed pleural empyema. This was the first study associating pre-hospital ibuprofen use in CAP with the development of empyema [48]. Consistently with these findings, a retrospective study conducted in 2 French hospitals reported an increasing incidence of complicated pneumonia, defined as a pleural effusion and/or a lung cavitation in children [49]. Between 1995 and 1999, 3% of hospitalized cases of pneumonia were complicated when compared with 23% in 2003. Multivariate analysis identified ibuprofen exposure as the only pre-hospital treatment independently associated with a higher risk of empyema or lung abscess [49]. Interestingly, this study also correlated the increased prevalence of complicated pneumonia in France with rising sales of liquid ibuprofen and supported these data with supplementary analysis [49]. In a British prospective cohort of 160 children hospitalized for CAP between 2009 and 2011, 40 developed a pleural empyema. Pre-hospital NSAIDs exposure was as high as 82% in these children who developed pleural empyema, compared with 46% in uncomplicated cases [50].

More recently, a prospective single-center study conducted in Poland including 203 children hospitalized for CAP between 2012 and 2014 evaluated the cumulative effect of ibuprofen dosing [51]. The authors demonstrated that pre-hospitalization higher cumulative dose of ibuprofen was associated with a 2.5 greater risk of pneumonia complications [51]. Forty-two children developed a pleural or pulmonary complication including para-pneumonic pleural effusion, pleural empyema, necrotizing pneumonia, and lung abscess. The exposure to a cumulative dose of ibuprofen higher than 78 mg/kg was associated with an increased risk of pleural or pulmonary complication [51]. Most of these pediatric case-control studies share the methodological weakness of not accounting for protopathic bias that occurs when it is not possible to determine whether the exposure to the factor preceded or not the occurrence of the complication. Hence, NSAIDs could have been prescribed because of symptoms (thoracic pain, fever) related to the beginning of a complication (parapneumonic pleural effusion, pleural empyema), rather than representing a concrete risk factor for the occurrence of a pleural or a pulmonary complication. In order to account for this bias, a case-control study was conducted in 15 French centers between 2006 and 2009 [24]. The cases involved consecutive children hospitalized for a pleural empyema occurring in the 15 days following a viral-associated respiratory tract infection treated at home. The controls were children with a viral-associated respiratory tract infection treated at home who did not require hospitalization and were matched for the general practitioner, age, viral symptoms, and season [24]. Eighty-three case-control pairs were studied. Infection was localized in the lower respiratory tract in 23% of cases and 34% of controls (*p* = 0.21). NSAIDs exposure, almost exclusively to ibuprofen, was involved in 39% of cases and 27% of controls (*p* = 0.08). Half of the children received paracetamol (47% vs 49%; *p* = 0.79), while 8% of the cases and 15% of the controls received antibiotics concomitantly to the first day of viral symptoms (*p* = 0.21). In a multivariate analysis, NSAIDs treatment starting during first 3 days of viral symptoms and administered for at least 1 day was independently associated with a higher risk of pleural empyema (OR = 2.79; 95% confidence interval (CI), 1.40–5.58). An antibiotic therapy started within the first 3 days of viral symptoms and administered for at least 6 days was independently associated with a lower risk of pleural empyema (OR = 0.32; 95% CI, 0.11–0.97). In the sub-group of children exposed to NSAIDs, the risk of pleural empyema was increased if the duration of antibiotic therapy was less than 6 days (OR = 3.01; 95% CI, 1.52–5.95) [24]. Finally, Meganathan et al., identified in a multivariate regression logistic analysis ibuprofen as an independent risk factor for the development of parapneumonic effusion/empyema in 30 children with CAP, (adjusted OR 6.8; 95%CI: 1.07–43.6) [52]. Table 3 summarizes all the evidences associating ibuprofen consumption with risk of developing complicated CAP.

**Table 3:** Ibuprofen exposure and pneumonia in children

1st Author, Year (Ref)	Population; Country	Study Design	Outcome	Key results	Comments
*Byington, 2002* [48]	540 children with CAP; US.	Retrospective cohort study	Risk factors for development of empyema in children with CAP.	Recent ibuprofen exposure was an independent risk factor of pleural empyema [OR 4.0 (95% CI 2.5 to 6.5)]	Timing of ibuprofen use relative to empyema development could not be established.
*Leroy, 2010* [47]	5182 children with CAP; France	Prospective, observational study	Severe bacterial infection in children who had received NSAIDs.	Frequency of hospitalization for severe bacterial infection as a possible adverse effect of NSAIDs use was 0.6% (95% CI 0.4 to 0.9).	No established causal relationship.
*François, 2010* [49]	767 children with CAP; France	Retrospective cohort study	Factors associated with development of empyema and lung abscess among children with CAP.	Recent ibuprofen exposure was an independent risk factor of pleural empyema [OR 2.57 (95% CI 1.51 to 4.35)].	‘Protopathic effect’ cannot be excluded.
*Elemraid, 2015* [50]	160 children presenting to with radiologically confirmed pneumonia; UK	Prospective, observational study	Risk factors for development of empyema requiring intervention in children with pneumonia	Children with empyema were more frequently assuming ibuprofen prior to hospital admission (82% vs 46.2%) OR 1.94 (97.5% CI 0.8 to 3.18).	Very high incidence of empyema (40/160, 25%)
*Le Bourgeois, 2016* [24]	83 children presenting with empyema compared with 83 controls with acute viral infection; France	Prospective, case-control study	Risk of developing empyema in children assuming NSAIDs during an acute viral infection	Recent NSAID exposure was an independent risk factor of pleural empyema [OR 2.8 (1.4–5.6)]	The risk of empyema associated with NSAIDs exposure was greater for children not assuming antibiotics
*Krenke, 2018* [51]	203 consecutive children with CAP; Poland.	Prospective, observational study	CAP risk in children assuming NSAIDs	A dose–effect relationship was found: exposure to a cumulative dose of ibuprofen higher than 78 mg/kg was significantly associated with an increased risk of pleuropulmonary complications, such as parapneumonic pleural effusion, pleural empyema, necrotizing pneumonia and pulmonary abscess [OR 2.5 (1.3–4.9)]	Higher cumulative dose of ibuprofen was associated with 2.5-fold higher OR for CAP complications.
*Meganathan, 2019* [52]	148 children with severe CAP; India	Prospective, case-control study	Predictor of CPE/ empyema in children (2–59 months) with CAP	NSAID use: OR 4.36 (95% CI 1.86 to 10.23); *p* = 0.007.	Disease spectrum bias

*CAP* Community-acquired pneumonia, *CPE* Complicated parapneumonic effusion, *NSAIDs* Non-steroidal anti-inflammatory drugs

In conclusion, pre-hospital administration of ibuprofen is associated with an increased risk of complicated lung infections in children, including empyema. Pediatricians should be aware of these possible complications and possibly avoid the administration of ibuprofen in the setting of febrile children with a suspicion of LRTI.

### Cystic fibrosis infectious exacerbations

Differently, from the above-discussed data the use of ibuprofen in children with cystic fibrosis seems to be safe and efficacious. As well known, cystic fibrosis (CF) is a genetic disease characterized by chronic lung inflammation and recurrent pulmonary infections. Pulmonary infections during cystic fibrosis disease course tend to be polymicrobial and are responsible for acute inflammatory response with an abundance of neutrophils, challenging the ability of the pulmonary system to clear them [53]. Ibuprofen use during CF pulmonary infections has been demonstrated to be effective and safe in containing the inflammatory response and helping the resolution of the infective episodes in numerous trials as well as in in vitro studies [53–58]. Already in 1995, Konstan and colleagues performed randomized controlled trial including both adults and children affected by CF. [53] The enrolled patients were randomly assigned to receive orally ibuprofen or placebo, twice a day for 4 years. Patients assigned to the ibuprofen group demonstrated a significant slower annual rate of change in FEV1 and a higher weight when compared to the patients assigned to placebo [53]. In 2007 Lands et al. reported the data of a pediatric double-blinded, placebo-controlled trial on 142 CF children randomized to receive either high-dose ibuprofen (20 to 30 mg/kg/twice daily) or placebo for a 2-year period. Children in the high-dose ibuprofen group exhibited a significant reduction in the rate of decline of forced vital capacity percent predicted (*p* = 0.03), but not FEV1% [57]. The ibuprofen group also spent fewer days in hospital after adjusting for age (1.8 vs 4.1 days per year; *p* = 0.07). No differences in serious adverse events were observed between the 2 groups [57]. In 2007 Lands and colleagues published the first Cochrane-review on the efficacy of oral non-steroidal anti-inflammatory drug therapy for lung disease in cystic fibrosis, further updated in 2013, 2017 and lately in 2019 [58–61]. The last published in 2019 identified 17 trials, but only 4 were finally included in the analysis. The authors concluded that high-dose ibuprofen could slow the progression of lung disease in patients affected by CF, especially in children [61]. Regarding the mechanism, a recent paper proposed that ibuprofen’s effectiveness in this setting might occur due to its antimicrobial effects against *Pseudomonas aeruginosa* and Burkholderia bacteria, 2 of the most fearsome pathogens associated with CF. [62] As a matter of fact, ibuprofen was able to reduce the growth rate and bacterial burden of these bacteria in a dose-dependent fashion in an acute pseudomonas pneumonia mouse model [62].

In conclusion, ibuprofen use in the setting of CF has been proven to be efficacious and safe in slowing down lung disease progression, thus strongly recommending its administration to face CF exacerbations.

### COVID-19

Coronavirus disease 2019 (COVID-19) is the result of a zoonotic infection caused by a novel coronavirus, severe acute respiratory syndrome coronavirus-2 (SARS-CoV-2) [63]. On March 11, 2020, the World Health Organization (WHO) officially declared COVID-19 a pandemic disease [64]. As of November 14th, 2020, SARS-CoV-2 spread in more than 200 countries worldwide and infected over 53 million people with 1.3 million deaths (https://www.worldometers.info/coronavirus/). Current experience suggests that adults are more susceptible to SARS-CoV-2 than children [65]. In adults, COVID-19 is typically characterized by severe interstitial pneumonia and hyper activation of the inflammatory cascade [66, 67]. Data from individual countries and several studies suggest that children under the age of 18 years represent about 8.5% of the reported cases, with relatively few deaths compared to other age-groups. Infection in children generally causes mild disease, and serious illness due to COVID-19 is seen only infrequently [68–73]. The most common findings amongst symptomatic children are fever (50%) and cough (38%). Shortness of breath, sore throat, rhinorrhea, conjunctivitis, fatigue, and headache are other commonly reported symptoms. Diarrhea, vomiting, and abdominal pain are common gastrointestinal symptoms that may be present with or without respiratory symptoms [68–73]. The basis of the decreased severity of SARS CoV-2 infection in children is still not well understood. Many hypotheses have been formulated including an immature receptor system, specific regulatory mechanisms in the immune system and cross-protection by antibodies directed towards common viral infections in infancy [74]. Particularly, most of the attention has been focused in possible differences in the expression, distribution and/or functioning of the human cell receptor expressing angiotensin-converting enzyme 2 (ACE2). As further elucidated, the binding of ACE2 is a crucial mechanism in SARS-CoV-2 pathogenesis. SARS-CoV2 structure includes 4 structural proteins: spike (S), membrane (M), envelope (E), and nucleocapsid (N). The spike protein binds to ACE2 and results in membrane fusion via conformational changes in the cell membrane [75, 76]. This process affects target organs *(lungs, digestive tract, heart, blood vessels, and kidneys)* where ACE2 expression is very high and induces local and systemic inflammatory responses involving the affected organ [75, 76]. It has been hypothesized that ACE2 expression may be decreased in children, although this has not yet been demonstrated. In addition, a greater dysregulation/dysfunction of both adaptive and innate immune responses and greater incidence of comorbidities in adults may also contribute to the more severe manifestations observed in adults vs children. Indeed, it seems pretty clear that the ‘cytokine storm’ occurring in the second phase of COVID-19 course is responsible of worsening of clinical symptoms. The molecular mechanisms underlying the altered pathological inflammation in COVID-19 are largely unknown [77]. Recently Sohn et al. reported that toll-like receptor (TLR) 4-mediated inflammatory signalling molecules are upregulated in peripheral blood mononuclear cells (PBMCs) from COVID-19 patients [78]. The blockade of TLR signalling through molecular checkpoints may contribute to developing a potential target treatment [79]. This inflammatory cascade seems to be infrequent in children, if we exclude the recently described cases of multisystem inflammatory syndrome [80–82]. The so-called MIS-C (multisystem inflammatory syndrome in children) is characterized by a hyperinflammatory shock, exhibiting similar features to atypical Kawasaki disease without significant respiratory issues in children previously exposed to SARS-CoV-2 [80–82]. The pathogenesis of this rare complication of COVID-19 in children may represent the equivalent of severe SARS-CoV2 induced cytokine storm in adults.

Due to the crucial role exerted by ACE2 expression in COVID-19 pathogenesis, Fang and colleagues at the beginning of the pandemic on March 2020 hypothesized a possible deleterious role of ACE2-stimulating drugs and ibuprofen on the course of SARS-CoV-2 infected patients [83]. Indeed, ibuprofen has been shown to exert significant effect on mice cardiac fibrosis increasing the level of expression of level of expression of ACE2 in a rat model of diabetes [84]. The increase of ACE2 expression may lead to a potential rise of SARS-CoV-2 viral load and consequently to a more severe disease course [83]. Therefore, the authors concluded their commentary discouraging the use of these drugs in the setting of COVID-19, although no evidence suggests a direct interaction between ibuprofen and ACE2 in humans. Nevertheless, this plausible mechanism together with the above-reported evidences of ibuprofen mediated worsening of LRTI, led the ANSM releasing a warning, asking whether patients showing symptoms of COVID-19 should use paracetamol rather than ibuprofen [85]. This warning was echoed by the British Medical Journal [86–88], causing a drop of 80% ibuprofen prescriptions in France [89]. The UK Medicines and Healthcare products Regulatory Agency (MHRA) reported that in the absence of clear evidences, patients should be advised to take paracetamol to treat the symptoms of COVID-19, unless paracetamol is not suitable for them [90]. In a similar way the European Medicine Agency (EMA) released the following statement: “There is currently no scientific evidence establishing a link between ibuprofen and worsening of COVID-19. EMA is monitoring the situation closely and will review any new information that becomes available on this issue in the context of the pandemic” [91]. Differently, the World Health Organization (WHO), after advising not to use ibuprofen for COVID-19, quickly retracted the public advisory on March 18, 2020 [92]. While the scientific debate whether to use or not ibuprofen in the course of COVID-19 continues [93–96], the first data have been published. Abu Esba and colleagues prospectively recruited 503 adults with a confirmed SARS-CoV2 infection of whom 40 (8%) using ibuprofen during the infection, 17 (3.4%) assuming other NSAIDs and 96 (19%) being chronically treated with NSAIDs before and during the infection. Neither the acute nor the chronic use of NSAIDs resulted to be associated with increased mortality or severe COVID19 [97]. More recently, Kragholm and colleagues reported the data of a retrospective, nationwide-based cohort study, including 4002 adults with COVID-19 of whom 264 (6.6%) treated with ibuprofen [98]. No significant association between ibuprofen prescription claims and severe COVID-19 was found [98]. Finally, Rinott and colleagues retrospectively evaluated the use of ibuprofen versus paracetamol during the course of SARS-CoV2 infection in 403 adult patients, confirming that ibuprofen was not associated with severe COVID-19 [99]. Up to date no study has been conducted in the setting of pediatric patients.

In conclusions, despite the initial warning, a causal link of a harmful effect of ibuprofen in patients with COVID-19 has not been established. Nevertheless, considering the overall uncertainty, the little amount of the published data and the milder course of pediatric COVID-19, we suggest to use acetaminophen monotherapy as first-antipyretic in children infected with SARS-CoV2. Further, well-designed studies are urgently needed in order to clarify this important issue and allow an improvement of cares for SARS-CoV2 infected patients.

## Conclusions

During the last decade the progressive widespread of ibuprofen administration in pediatric diseases led to several concerns on possible serious side effects, including the worsening of infectious processes. This narrative review clearly underlines that there are sufficient evidences to contraindicate ibuprofen for the management of chickenpox symptoms, due to the elevated risk of NSTI. Despite the lack of well-conducted trials, several papers suggest that pre-hospital use of ibuprofen may increase the risk of complicated pneumonia in children. Thus, we recommend caution on its administration in the febrile children with a suspicion of LRTI. Differently, ibuprofen’s efficacy and safety in the setting of cystic fibrosis is corroborated by RCTs and metanalyses and it is therefore strongly recommended. Conflicting data have been published for the management of the septic children. Up to date it is not possible to draw any conclusion and further well-designed trials are urgently warranted. Finally, the COVID-19 pandemic raises many questions regarding ibuprofen administration during the acute respiratory distress syndrome caused by SARS-CoV2. The first published papers seem to be reassuring at least in adults. However, while waiting for real-life pediatric data taking into account the milder course of SARS-CoV2 infection in children, the risks of bacterial superinfection and the above reported data on LRTI, we recommend continuing use paracetamol as first choice in the course of COVID-19.

## Data Availability

Not applicable.
